# Bortezomib induces methylation changes in neuroblastoma cells that appear to play a significant role in resistance development to this compound

**DOI:** 10.1038/s41598-021-89128-0

**Published:** 2021-05-10

**Authors:** Karolina Łuczkowska, Katarzyna Ewa Sokolowska, Olga Taryma-Lesniak, Krzysztof Pastuszak, Anna Supernat, Jonas Bybjerg-Grauholm, Lise Lotte Hansen, Edyta Paczkowska, Tomasz K. Wojdacz, Bogusław Machaliński

**Affiliations:** 1 Department of General Pathology, Pomeranian Medical University, Powstańców Wlkp. 72, 70-111 Szczecin, Poland; 2 Independent Clinical Epigenetics Laboratory, Pomeranian Medical University, Unii Lubelskiej 1, 71-252 Szczecin, Poland; 3 Laboratory of Translational Oncology, Intercollegiate Faculty of Biotechnology, Medical University of Gdańsk, Dębinki 1, 80-211 Gdańsk, Poland; 4 Department of Algorithms and Systems Modelling, Faculty of Electronics, Telecommunications and Informatics, Gdańsk University of Technology, Narutowicza 11/12, 80-233 Gdańsk, Poland; 5 Department for Congenital Disorders, Statens Serum Institut, Artillerivej 5, 2300 København S Copenhagen, Denmark; 6 Department of Biomedicine, Aarhus University, Hoegh-Guldbergsgade 10, 8000 Aarhus, Denmark; 7 Aarhus Institute of Advanced Studies, Hoegh-Guldbergs Gade 6B, 8000 Aarhus, Denmark

**Keywords:** Cancer, Molecular biology, Neurology, Oncology

## Abstract

The anticancer activity of bortezomib (BTZ) has been increasingly studied in a number of indications and promising results for the use of this treatment have been shown in neuroblastoma. As BTZ treatment is usually administered in cycles, the development of resistance and side effects in patients undergoing therapy with BTZ remains a major challenge for the clinical usage of this compound. Common resistance development also means that certain cells are able to survive BTZ treatment and bypass molecular mechanisms that render BTZ anticancer activity. We studied the methylome of neuroblastoma cells that survived BTZ treatment. Our results indicate that BTZ induces pronounced genome wide methylation changes in cells which recovered from the treatment. Functional analyses of identified methylation changes demonstrated they were involved in key cancer pathology pathways. These changes may allow the cells to bypass the primary anticancer activity of BTZ and develop a treatment resistant and proliferative phenotype. To study whether cells surviving BTZ treatment acquire a proliferative phenotype, we repeatedly treated cells which recovered from the first round of BTZ treatment. The repetitive treatment led to induction of the extraordinary proliferative potential of the cells, that increased with subsequent treatments. As we did not observe similar effects in cells that survived treatment with lenalidomide, and non-treated cells cultured under the same experimental conditions, this phenomenon seems to be BTZ specific. Overall, our results indicate that methylation changes may play major role in the development of BTZ resistance.

## Introduction

The most common and fetal tumour in children is neuroblastoma (NB)^1^. This neuroendocrine tumour originates from the sympathoadrenal lineage derived from the neural crest, and displays very heterogeneous clinical behavior ranging from chemo-resistant and aggressive to even spontaneously regressing tumours^2^. Overall survival of patients with relapsed refractory neuroblastoma is still less than one year^3^. However, 20% of neuroblastoma patients survive 5 years after first relapse, moreover the outcomes of the disease greatly depend on the age of diagnosis and time to relapse, suggesting that there is a subset of the patients that can benefit from personalized treatment^3–6^.

Bortezomib (BTZ) is a selective inhibitor of 26S proteasome, a protein complex degrading unneeded or damaged intracellular proteins. The mechanism of the BTZ antitumor activity is not yet fully understood but inhibition of the proteasome prompts tumour cells to apoptosis and neoplastic cells have been shown to be more dependent on proteasome inactivation than normal cells^7^. BTZ is used as first line treatment in multiple myeloma and its activity against solid tumours has already been demonstrated^8^. In one of the first clinical trials exploring the utility of this compound in the treatment of neuroblastoma, BTZ in combination with irinotecan was shown to be well tolerated and displayed promising activity in patients with relapsed/refractory high-risk neuroblastoma^9^. Two other clinical trials are currently exploring the use of this drug in neuroblastoma treatment (NCT00644696 and NCT02139397).

A major challenge of treatment with BTZ is the development of treatment resistance and side effects such as peripheral neuropathy. As BTZ is administered in cycles the resistance develops over time in the majority of patients. The fact that these patients develop treatment resistance, indicates that some cancer cells are able to bypass anticancer activity of the BTZ.

Here, we compared the methylomes and the proliferative potential of cells that recovered from treatment with BTZ to cells recovered from the treatment with lenalidomide and non-treated cells but cultured under the same experimental conditions. Our data show that only BTZ induces genome wide methylation changes, which appear to be involved in the development of a therapy resistant phenotype. Moreover, we show that cells repeatedly treated with BTZ acquire significant proliferative potential.

## Materials and methods

### Cell culture set up

**Figure 1:**
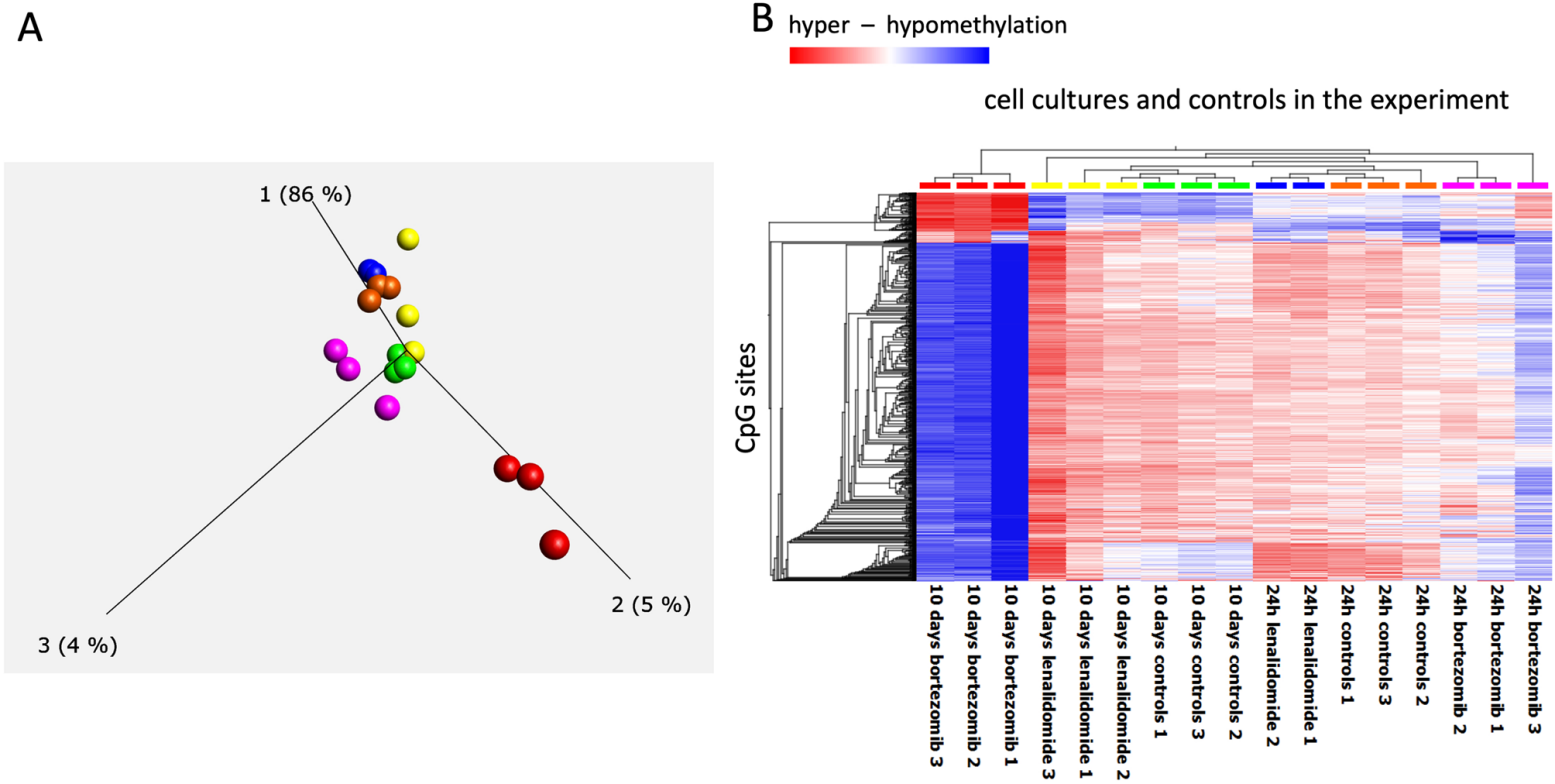
PCA (Principle Component Analyses)—(**A**) and heatmap—(**B**), illustrating results of genome wide methylation changes in cells under treatment and controls. PCA indicates that genome wide methylation profile of cells recovered from BTZ treatment after 10 days (red color -three replicates) have significantly different methylation profile than cells in other cultures in the experiment including: yellow—cells recovered from treatment with lenalidomide (after 10 days), green—controls after recovery, blue—cells 24 h after treatment with lenalidomide, orange—24 h treatment controls, pink—cells 24 h after treatment with BTZ. Heatmap illustrating unsupervised clustering analyses of methylation changes at the most informative CpG sites in the data set verifies PCA analyses with cells recovered after BTZ treatment (red color top of chart) displaying significant hypomethylation (blue color in the heatmap) and a degree of hypermethylation (red color in the heatmap) in comparison to other cell cultures in the experiment.

We used SH-SY5Y neuroblastoma cells (human, ECACC; Sigma Aldrich, St. Louis, MO, USA) to study the effects of BTZ (Cell Signalling Technology, Danvers, MA, USA) and lenalidomide (Cayman chemical, Ann Arbor, MI, USA) treatment. The cells were cultured in serum-free conditions in Minimum Essential Medium (MEM) (Sigma Aldrich, St. Louis, MO, USA) and Ham's F-Nutrient Mixture (Thermo Fisher, Waltham, MA, USA) (mixed in ratio 1:1) medium supplemented with L-glutamine (2 mM) and streptomycin (100 μg/mL), penicillin (100U/mL) without fetal bovine serum (FBS) at 37 °C in saturated humidity atmosphere containing 5% CO_2_. The medium in the cell cultures was replaced every 2–3 days during the experiment. The outline of the experimental set up for one of the treatments is shown in Supplementary material 1, Fig. 1. Specifically, after the initial cultures for each of the treatments and non-treatment control were established (cell density 2 × 10^6^ cells/well), medium containing BTZ (50 nM/L) or lenalidomide (10 μmol/L) was administered to the cells cultures. Non-treated cells were passaged the same way as treated cells and used as experimental control. Medium containing BTZ or lenalidomide was removed after 24 h. The cells after each treatment (along with the non-treated control) were divided and half of the cell cultures were left to recover for 12 days before next round of treatment, whilst the second part of the cultures were seeded on six plates with 18 technical replicates each for the cell proliferation potential tests. The cell proliferation was tested on days 0, 2, 5, 7, 9 and 12 after each treatment using one of the plates with 18 technical replicates of treated cells as shown in Fig. 1. The manufacturer of the MTT assay kit recommends 12 replicates for cell proliferation (see below for description), however we set up our experiment to include 18 measurements of cell proliferation at each time point.

### DNA extraction

DNA was isolated from three separate cell incubations for each group. Total DNA was isolated from SH-SY5Y cells (1.5 × 10^6^) using NucleoSpin TriPrep (Macherey Nagel, Düren, Germany) following the manufacturer's instructions. The DNA concentration and quality of obtained were assessed by Epoch spectrophotometer (Biotek, Winooski, VT, USA).

### Cells proliferation assay

To test cell proliferative potential, we used MTT (3-(4,5-dimethylthiazol-2-yl)-2,5-diphenyltetrazolium bromide) assay kit (Abcam, Cambridge, UK, cat. ab211091) and the tests were performed according to manufacturer protocol. This cell proliferation assay is based on the conversion of water soluble and colourless MTT (3-(4,5-dimethylthiazol-2-yl)-2,5-diphenyltetrazolium bromide) to purple insoluble formazan. During the procedure cells are incubated with 50 μL of MTT for 3 h to allow cells to convert MTT to insoluble formazan crystals. Subsequently detergent is added to lyse the cells and solubilize the crystals of the insoluble formazan which have a purple colour*.* Then the concentration of the dissolved insoluble formazan is measured using Varioskan LUX multimode microplate reader (TSA, Thermo Fisher Scientific, Waltham, MA, USA) with OD 590 nm. The amount of absorbed light is proportional to the number of viable cells^10^.

### Genome wide methylation analyses

We used the Infinium MethylationEPIC Beadchip array, Illumina (EPIC array), to screen for methylation changes in treated cells. This chip allows assessment of methylation status at more than 850 000 CpG loci. The raw microarray data was processed using ChAMP pipeline with default data processing settings^11^. After raw data processing and QC, the dataset contained methylation data (beta values) for a total of 725 096 CpG sites. Subsequent data analyses was performed using Qlucore Omics Explorer (Qlucore, Lund, Sweden) and included projection score based variance filtering^12^. This type of data filtering allows one to compute a consensus and the most informative variable subset for the downstream analyses. We evaluated the projection score during variance filtering (variance defined as the square of the standard deviation: σ^2^ of the variable) and selected the data set for which we could achieve the highest projection score indicating the most informative variable list. The data in the consensus data set after variance filtering were corrected for multiple testing. To evaluate the functional context of the observed methylation changes we used GREAT (Genomic Regions Enrichment of Annotations Tool) version 4.0.4 tool, which allows to predict functions of cis-regulatory regions. For identification of the DMRs,—ChAMP platform and lasso algorithm^11^ and HOMER v4.1 were used to analyse the influence of the methylation changes on the transcription factor binding suites^13^.

### Validation of the results of the microarray results

To follow good laboratory practice, we validated the methylation changes observed using EPIC array with Methylation-Sensitive High-Resolution Melting (MS-HRM)^14^. Briefly 500 ng of DNA were subjected to sodium bisulfite modification (EZ-96 DNA Methylation-Gold kit, Zymo Research, Irvine, CA, USA) according to the manufacturer’s instructions. MS-HRM assays targeting five of the DMR identified in microarray experiments were designed as described by Wojdacz et al.^14,15^. MS-HRM analyses were performed using the LightCycler480 platform (Roche, Mannheim, Germany) with a PCR reaction mixture consisting of 2 × EpiMelt MS-HRM Master Mix (MethylDetect ApS—Ampliqon A/S, Denmark), 500 nM of each primer, and 10 ng of bisulfite-modified DNA (theoretically calculated concentration) in a final volume of 10 μL. For qualitative methylation assessment, we included standard samples in each assay obtained from artificially methylated DNA (Universal Methylated Human DNA Standard, Zymo Research, Irvine, CA, USA) and artificially non-methylated DNA (Epitech Control DNA, Qiagen, Hilden, Germany). Contact us for technical specifications of the MS-HRM assays.

## Results

### Cells surviving bortezomib treatment acquire significant methylation changes

Firstly, we compared genome wide methylation profiles of cells that; recovered from BTZ treatment, (and within 10 days re-established cell culture) with non-treated cells and cells treated with lenalidomide. As shown by PCA plots (Fig. 1A) the methylation levels at the 1614 most informative CpG sites in this data set were almost identical in cells from the nontreated control culture, both after 24 h and 10 days of the culture.

Similarly, the methylation profiles of the cells from the culture treated with lenalidomide and also harvested 24 h and 10 days after treatment did not change (Fig. 1A). However, the methylation profile of the cells harvested 10 days after treatment with BTZ was significantly different from all other cells, with 86% variance at first component in that data set attributed to those differences (Fig. 1A). The unsupervised clustering of this data further verified that BTZ treatment induced genome wide methylation changes in the methylome of the cells that recovered from the treatment (Fig. 1B).

### BTZ induces both CpG specific and regional methylation changes

**Figure 2:**
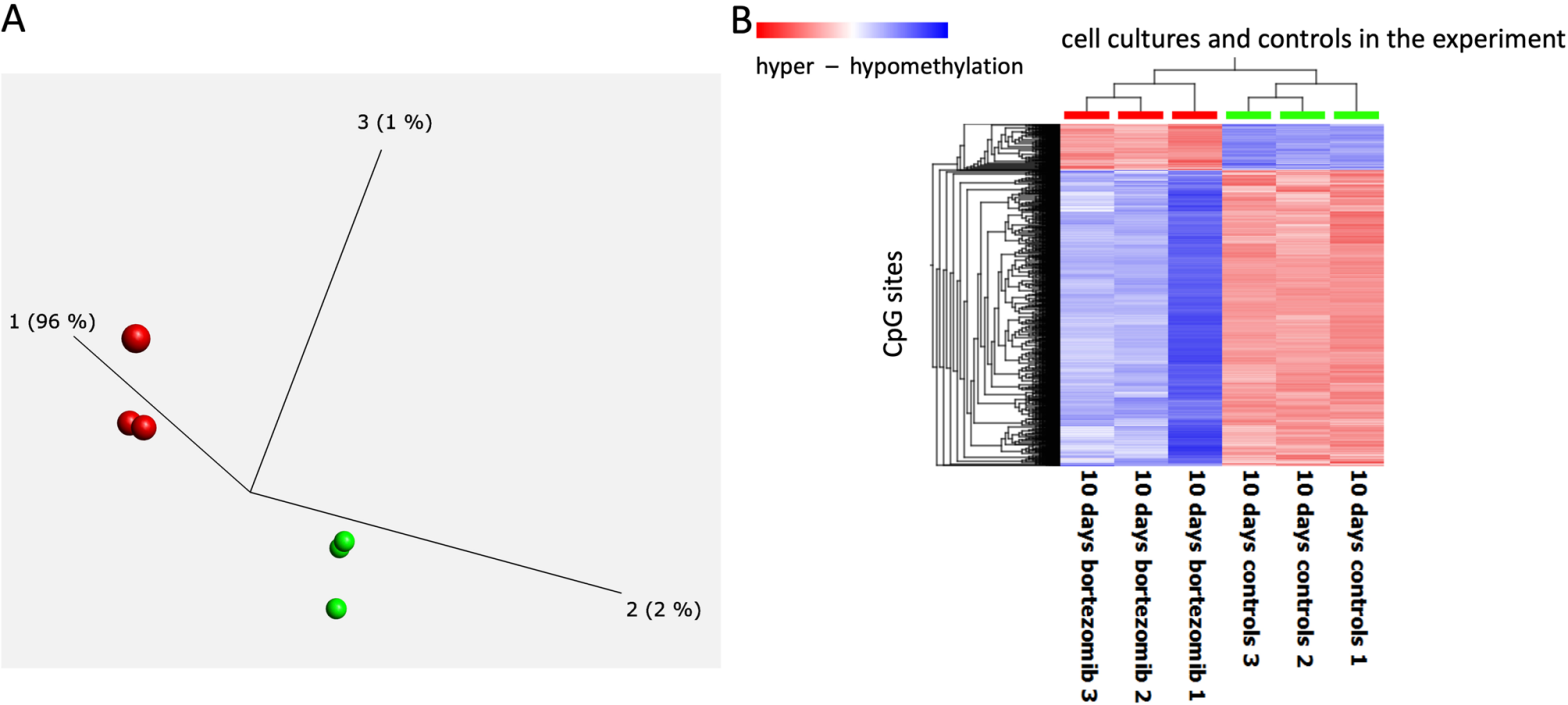
PCA (Principle Component Analyses)—(**A**) and heatmap—(**B**), illustrating results of genome wide methylation changes in cells under treatment and controls. PCA indicates three replicates of genome wide methylation profiles of cells recovered after BTZ treatment after 10 days (red color) have significantly different methylation profile than controls (green color). Heatmap illustrating unsupervised clustering analyses of methylation changes at the most informative CpG sites in the data set verifies PCA analyses with cells recovered after BTZ treatment (red color top of chart) displaying significant hypomethylation (blue color in the heatmap) and a degree of hypermethylation (red color in the heatmap) in comparison to controls (green, top of chart).

We further analysed methylation changes observed between cells recovered from BTZ treatment and control cells from the non-treated culture. Overall, BZT treatment induced more than 10% methylation level changes at 4142 CpG sites across the genome (the list of the CpG sites can be found in Supplementary Material 1). The first component in the PCA analyses of these data showed that methylation changes at this set of the CpG sites accommodated 96% of the variance in the data (Fig. 2A) and unsupervised clustering further corroborated those findings (Fig. 2B).

**Figure 3:**
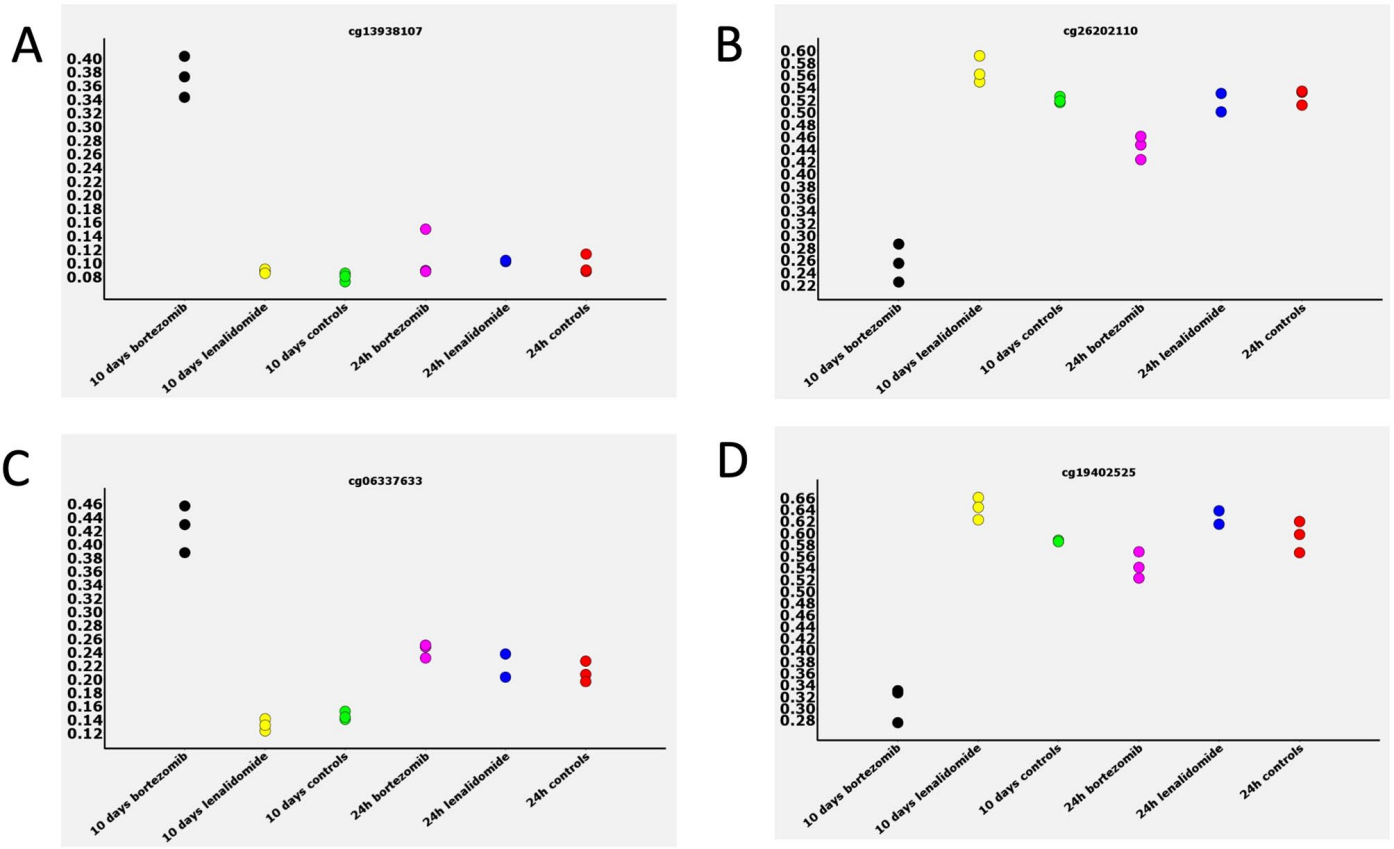
Examples of the methylation changes observed at specific CpG sites across all the treatments and controls. The beta-values representing methylation levels are plotted at the vertical axis and the type of cell at the horizontal axis. Gain of methylation (hypermethylation) in the cells treated with BTZ and recovered from treatment was observed at CpG sites in (**A**) and (**C**), and loss of methylation at CpG sites in (**B**) and (**D**).

The majority of methylation changes observed were loss of methylation, with 563 and 3579 of the CpG sites, displaying hyper and hypomethylation, respectively. To confirm these findings, we compared beta-values representing methylation levels at specific CpG sites between all treatments and time points in the experiment for a random set of CpG sites. Figure 3 describes the representative results of that comparison with significant methylation changes observed at specific CpG sites between all cultures and time points in the experiment. Interestingly, the majority changes observed in the data set seem to involve loss or gain of methylation at around 50%, this may indicate that only one allele was affected by the methylation change.

**Figure 4:**
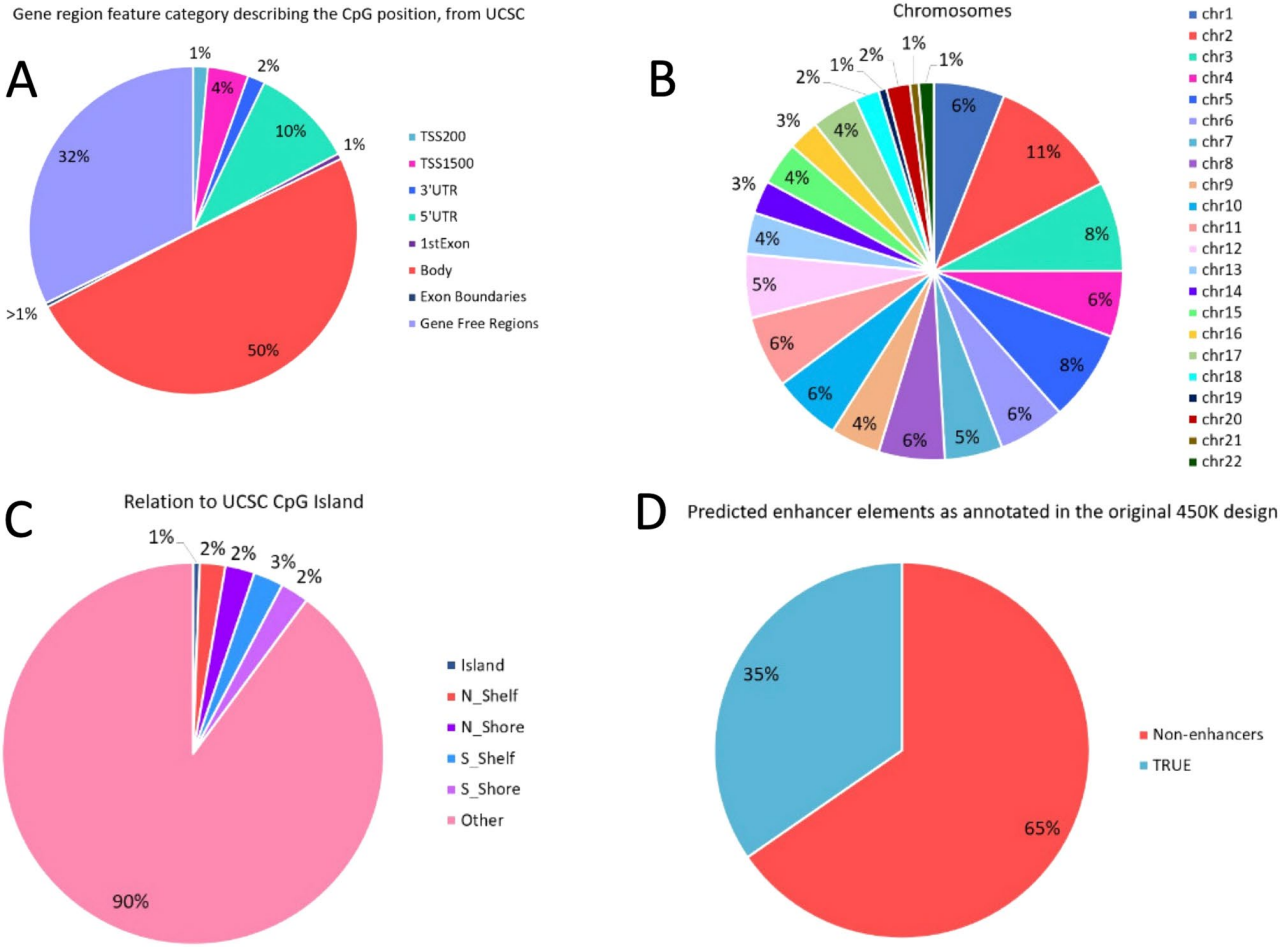
Genomic localization analyses of CpG sites differentially methylated in cells recovered after BTZ treatment and controls. (**A**)—according to the gene functional elements, (**B**)—at chromosomes, (**C**)—regarding CpG islands, (**D**)—enhancer occupation sites.

We then annotated the identified methylation changes and found that the majority occurred in gene bodies or gene free regions of the genome (Fig. 4A).

Changes did not colocalize to specific chromosomes (Fig. 4B) and generally 90% of them did not involve CpG-islands (Fig. 4C). However, more than one third of the changes involved enhancer elements (Fig. 4D). Apart from analysis of the genome wide methylation changes at single CpG sites, we also investigated whether BZT induced methylation changes that involved larger regions of the genome. The Probe Lasso algorithm used in this analysis identified 49 regions with altered methylation (Differentially Methylated Regions—DMR) (detailed annotation of this region can be found in Supplementary material 1). Following good laboratory practice, we designed five MS-HRM assays to validate the methylation changes at the identified DMRs. The MS-HRM results confirmed the bioinformatics analyses. The results from MS-HRM validation experiments are presented in (Supplementary Material 2).

### Functional context of the BTZ related methylation changes

**Figure 5:**
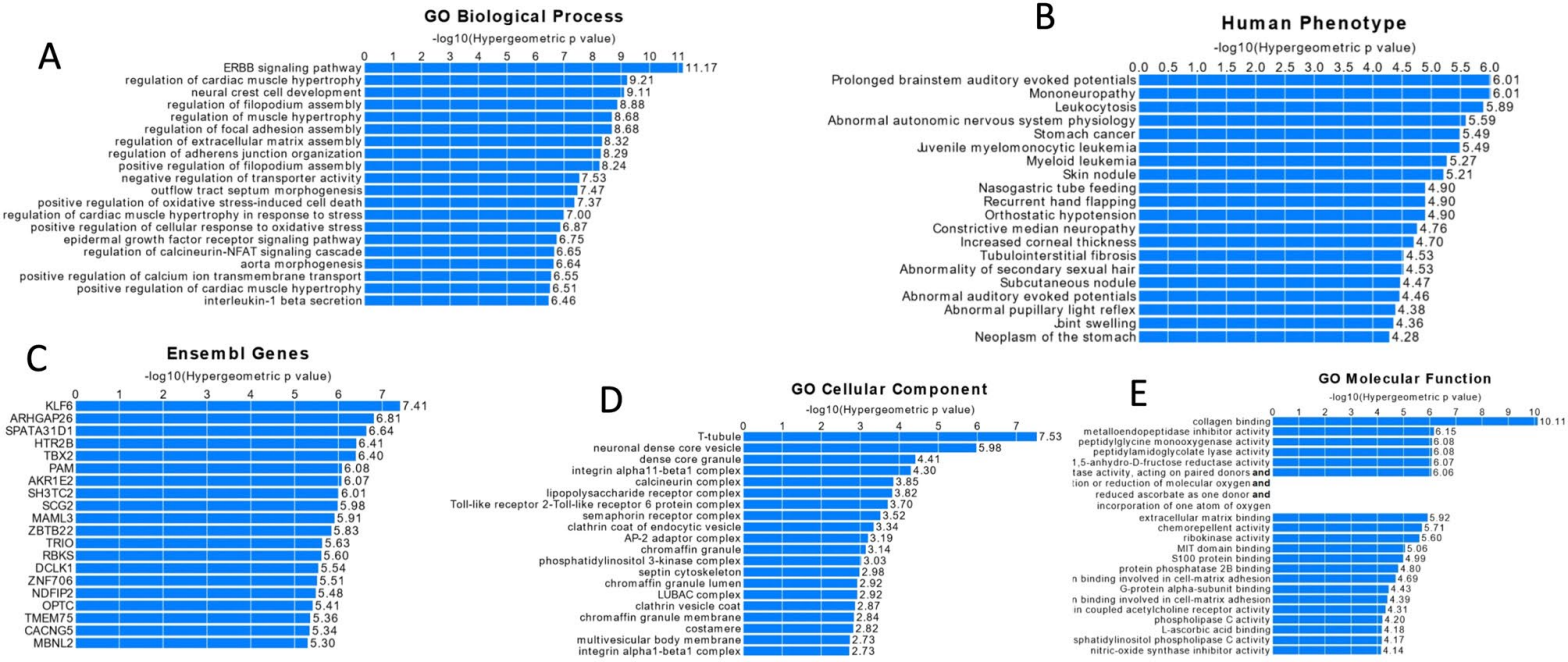
The output of GREAT analyses for five ontology terms linked to the methylation changes for five ontology categories: A—GO Biological Processes, B—Human Phenotype, C—Ensemble Genes, D—GO Cellular Component, E—GO Molecular Function.

As described in the above section the majority of identified methylation changes were not located in the promoter or obvious regulatory regions of the genome (e.g. CpG islands) for which the biological function is well annotated. The GREAT platform, allows to assign potential biological functions to the sets of non-coding genomic regions (e.g. regions at which methylation changes were observed) by analysing the annotation of those regions to the nearby genes. We used this platform to identify biological processes potentially affected by the BTZ induced methylation changes (Fig. 5). This analysis annotated methylation changes observed to five ontology categories: GO Biological Processes (Fig. 5A), Human Phenotype (Fig. 5B), Ensemble Genes (Fig. 5C), GO Cellular Component (Fig. 5D) and GO Molecular Function (Fig. 5E).

The top classification in each ontologies were processes potentially involved in drug-resistance and proliferation. Specifically, the biological pathway most significantly associated with identified methylation changes was the “ERBB signalling pathway” as well as processes critical for the function of cell microenvironment such as “regulation of filopodium assembly”, “regulation of focal adhesion assembly”, “regulation of extracellular matrix assembly—ECM” or “regulation of adhering junction organization”. Similar terms were significantly enriched in categories such as Go Cellular component and GO molecular function, further supporting the accuracy of these analyses. Disruption of the majority of the processes that were identified in GREAT analysis, can potentially result in the development of treatment resistance and increase of the proliferative potential of the cells.

### BTZ induces methylation changes at the transcription factor binding sites

The disruption of transcription factor (TF) binding is one of the molecular mechanisms involved in development of not only disease but also treatment resistance and side effects. We used HOMER base motif enrichment analyses to identify regulatory elements that are specifically enriched in the vicinity (± 200 bp) of the CpG sites at which we observed BTZ related methylation changes. This analysis showed that motifs most significantly enriched at those loci with; *p* > 1e−12, more than 20% target sequences associated with the motif and the highest motif score were, JunB (bZIP), GATA3 (Zf), NF1-halfsite (CTF) and Smad4 (MAD) (for details see Supplementary Material 2, Table S1). All TF families have been linked to the disease but more importantly, for example JunB (bZIP) has previously been shown to be essential for proliferation, survival and drug resistance of multiple myeloma and for the treatment of this disease where BTZ was initially developed^13^.

### Cells re-treated with BTZ acquire significant proliferative potential.

**Figure 6:**
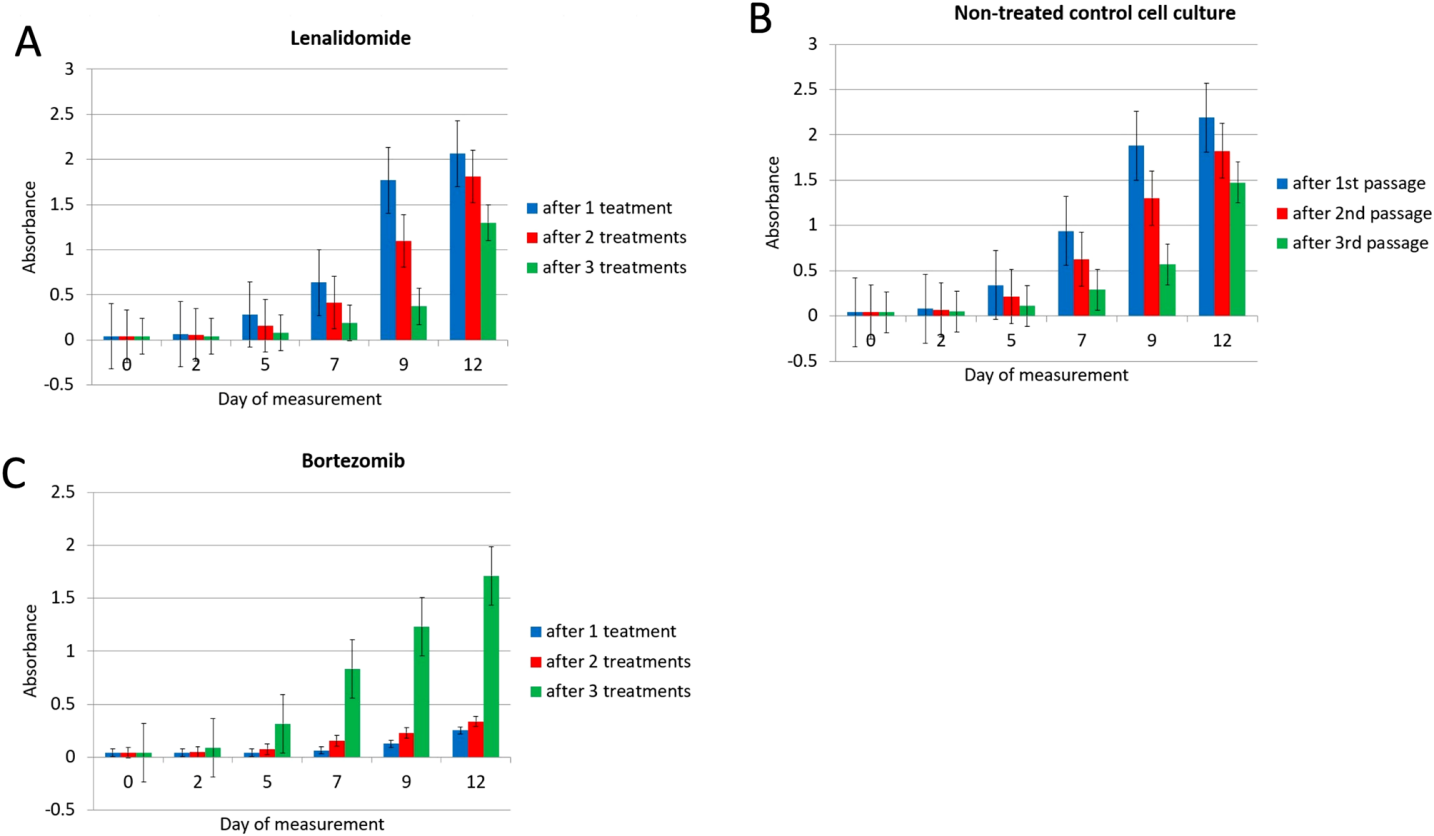
Proliferative potential analyses of cells (MTT assay). The “absorbance” measurement reflects the number of cells in each culture (see materials and methods) and was assessed in cells undergoing and recovered from one—blue, two—red and three—rounds of treatment. (**A**)—lenalidomide, (**B**)—non-treated controls and (**C**)—BTZ. The cell count increase was measured after 2, 5, 7, 9 and 12 days of the culture, see also Figure S1 for the graphical outline of this experimental set up.

Treatment with the BTZ is administered in cycles and the major challenge of this protocol is that patients undergoing increasing number of cycles develop treatment resistance. Addressing the biology of neoplastic disease, in principle this means that some cancer cells gradually adapt to anticancer activity of the BTZ and can proliferate even in the presence of this treatment compound. Our functional analyses of methylation changes induced by BTZ indicated that those alterations were likely to affect processes involved in development of a proliferative phenotype. Therefore, we studied the proliferative potential of cells repeatedly treated with BTZ. In this experiment, we measured proliferation of cells as they were recovering from the first treatment at 0, 2, 5, 7, 9 and 12 days and repeated the procedure for cells recovered after second and third treatment (detailed graphic description can be found in Supplementary material 2, Fig. 1). The experiment was controlled against treatment with lenalidomide and non-treated cell cultures that were passaged following the same procedure as cells under treatment. Figure 6 shows the relative increase of cells in each of the cultures following three rounds of treatment. The proliferation potential of cells after treatment with lenalidomide (Fig. 6A) and non-treated cells (Fig. 6B) did not increase but even markedly decreased after treatment rounds or passage alone (in the case of the non-treated cells). At the same time cells that recovered from second and third rounds of treatment (green bars—Fig. 6C) recovered quickly and divided almost exponentially. To validate these results, the experiment was repeated three times and each time only cells treated with BTZ showed an increase in proliferative potential.

## Discussion

BTZ resistance development is an important challenge with the clinical use of this compound, especially since BTZ anticancer activity is increasingly being reported in numerous indications, extending the use of this medication. It is unlikely that BTZ treatment induces genetic lesions that lead to drug resistance acquisition, thus we evaluated whether epigenetic mechanisms are involved in the development of BTZ resistance. We observed significant DNA methylation changes in cells that recovered from BTZ treatment, the changes included not only single CpG sites but also larger regions of the genome. Importantly, we did not observe these changes in methylome of cells used as experimental controls and cells under identical treatment protocol with lenalidomide.

We used bioinformatics and available data bases to assess the biological meaning of identified methylation changes. Those analyses linked BTZ induced methylation changes to several biological processes, which when disrupted may explain the acquisition of treatment resistance and proliferative potential in treated cells. The top hit in this analysis was the ERBB signalling pathway, playing an essential role in cell physiology including regulation differentiation, proliferation, survival, and migration^16,17^. The ERBB pathway signalling includes four tyrosine kinase transmembrane glycoproteins: epidermal growth factor (EGF), epidermal growth factor receptor (EGFR also known as ERBB1), ERBB2, ERBB3, and ERBB4^18^. ERBB family members overexpression has been shown to occur in various treatment resistant cancers including: gefitinib in head and neck squamous carcinoma cells^19^, gefitinib and erlotinib in lung cancer^20^, 5-fluorouracil, cytoxan, doxorubicin, taxol, and vinorelbin in breast cancer^21^. Moreover, BTZ has been shown to downregulate *ERBB2* and therefore inhibit important cell cycle entry and proliferation PI3K-Akt-mTOR and MAPK pathways^22,23^. As much as inhibition of those pathways maybe a mechanism of BTZ anticancer activity, bypassing of these processes via BTZ induced methylation changes may lead to an increased proliferative potential of cells. Other biological processes that we found affected by BTZ induced methylation changes were processes directly linked to the proliferative potential of cells including: “regulation of filopodium assembly”, “regulation of adhering junction organization” and “regulation of focal adhesion assembly” critical for extracellular matrix "ECM" functioning^24^. The "regulation of extracellular matrix assembly" was also one of the top terms in the biological processes analysis of identified methylation changes. Those processes involved in the regulation of extracellular matrix assembly are critical for cell microenvironment functioning, and have been shown to take part in regulation of cancer growth, invasion and acquisition of treatment resistance^25–27^. Interestingly, activity of extracellular matrix components have previously been linked to BTZ activity. For example, Multiple Myeloma cells incubated in medium containing two extracellular matrix proteins: hyaluronan and proteoglycan link protein 1 (HAPLN1) displayed increased levels of cell proliferation despite BTZ treatment when compared to the same cells incubated only with BTZ. This suggests that the extracellular HAPLN1 may be involved in developing resistance and suppress the anti-tumour effect of BTZ^28^.

Importantly, we observed similar significantly enriched terms across different ontological categories analysed. For example, “collagen binding” was a top hit in GO molecular function ontology (Fig. 5E) and collagens are one of the most important ECM proteins. The upregulation of collagen gene expression is common across the majority of cancers and involved in cell proliferation^29–34^.

The “positive regulation of cellular response to oxidative stress” was another biological process significantly associated with methylation changes. BTZ was shown to induce oxidative stress and reduce proliferation in BTZ-sensitive mantle cell lymphoma (MCL)^35^. At the same time, high basal antioxidant capacity was associated with resistance treatment and poor clinical outcome for patients treated with BTZ^35^. Whether methylation changes are involved in the increase of antioxidant capacity within resistant cells is yet to be investigated however, our results point in that direction.

The top gene (Fig. 4 C) linked to the identified methylation changes was *KLF6* (Krüppel-like factor 6). Up regulation of *KLF6* has already been linked to increased susceptibility to BTZ of myeloma cells^36^. Moreover, elevated level of *KLF6* expression were observed in MM patients who responded well to BTZ treatment^37^. These results suggest involvement of the processes regulated by *KLF6* in response to BTZ treatment and bridging of those process may result in a treatment resistant phenotype. Apart from this top hit, a number of genes shown to be involved in proliferation of cancer cells were statistically significantly associated with BTZ induced methylation changes, including: *ARHGAP26*^38^*, TBX2*^39,40^*, MAML3*^41^, *DCLK1*^42,43^. The “GO cellular components” (Fig. 4D) was another annotation term category identified by GREAT analyses. One of theh top hits in this analysis was “lipopolysaccharide (LPS) receptor complex”. Stessman et al., demonstrated that incubation of BTZ-resistant multiple myeloma cells with LPS restores the cells sensitivity to BTZ^44^. Another of the top hits in this analysis was “phosphatidylinositol 3-kinase complex”, which is part of the ”phosphatidylinositol 3-kinase/AKT (PI3K/AKT)” pathway, and addition of the PI3K/AKT inhibitor (BKM120) to the culture of the BTZ resistant myeloma cells increased the apoptotic effect and eliminated resistance to BTZ^45^. Finally, the enrichment analyses of ontology terms related to the human phenotype (Fig. 4B) and BTZ induced methylation changes showed strong association with terms linked with disorders within the nervous system such as mononeuropathy or abnormal autonomic nervous system physiology. Similar ontological categories linked to the nervous system were present in other ontological categories identified by the GREAT analysis, for example “neural crest cell development” (Fig. 4 A), “neuronal dense core vesicle” (Fig. 4 D), and genes such as *KLF6*^46^, *TBX2*^47^, *DCLK1*^48^ (Fig. 4C). This finding could be confounded by the cell line model used in our experiment. However, peripheral neuropathy is one of the most serious BTZ treatment side effect and although this requires further investigation, results may suggest methylation changes are involved in the development of BZT-induced neuropathy.

We then speculated that methylation changes induced by BTZ alter the binding of specific TF and therefore disrupt the above processes. To validate this hypothesis, we analysed the TF categories of TF that may be altered by BTZ related methylation changes. The most striking observation confirming the above assumption was the identification of JunB (bZIP), as the most significantly enriched motif I this analyses. This TF has very recently been shown to play an essential role in cell proliferation, survival and drug resistance and has been proposed as a target future multiple myeloma therapy^49^. Our results indicate that methylation changes at these TF binding sites may serve as underlying mechanism of these activities. The GATA3 (Zf) is another of the top hits from our analyses and BTZ treatment has shown to induce the redistribution of GATA3 (Zf) to the transcriptionally relevant regions^50^, as well as decrease occupancy of this and another TFs^51^. It is plausible to speculate that this activity can also be mediated by regulation of the accessibility of the GATA3 (Zf) transcription bindings sites. CREB1 TFs was shown to associate with RSK2 to regulate several signalling pathways and inhibition of RSK2 sensitized MM cells to BTZ. This is an example of the opposite effect to that observed in our cell proliferation experiment, nevertheless, we can speculate that methylation changes at this TF binding sites may be involved in this phenomenon^52^. In the performed literature searches, we did not identify studies describing a direct link between BTZ activity and NF1-halfsite (CTF), TEAD3 (TEA), Egr2 (Zf), Smad4 (MAD) and PROP1 transcription factors identified in these analyses. However, considering these findings, it is now worth exploring the potential effect of BTZ on those TFs, especially as they regulate various physiological processes, that have already been linked to disease and therefore may be potentially important in the process of acquisition of the BTZ resistance. Overall, this result indicates that methylation changes at the TF binding sites are likely to be a putative mechanism of BTZ resistance.

In summary, the major mode of BTZ anticancer activity is proteasome inhibition, causing imbalance between production and degradation of intracellular proteins. This activity leads to apoptosis of cancer cells. However, we have shown that a subset of cells can survive BTZ treatment, develop a proliferative phenotype, and are characterised by a significantly altered methylome. In those cells, the changes of methylation may play a significant role in development of BTZ adaptation, especially as we have been able to link identified methylation changes to physiological processes, which when bypassed may induce a proliferative phenotype. It should be noted that the identified methylation changes are potentially reversable and therefore are an attractive target for new treatment development. Additionally, compounds displaying activity towards disease related methylation changes are already approved for treatment use in certain cancers, modification of the current treatment regimens to include these compounds along with BTZ is an option worth investigating.

## Supplementary Information

Supplementary Information 1.

Supplementary Information 2.

## Data Availability

Microarray data described in this paper as accessible under: GSE171911.
