# Supplementary material for: Bortezomib induces methylation changes in neuroblastoma cells that appear to play a significant role in resistance development to this compound
Source: Sci Rep. 2021 May 10;11:9846. doi: 10.1038/s41598-021-89128-0 (PMC8110815; doi:10.1038/s41598-021-89128-0)
Supplement: Supplementary file 2 — Supplementary Information 2. [file 41598_2021_89128_MOESM2_ESM.docx]

**Bortezomib induces methylation changes in neuroblastoma cells that appear to play a significant role in resistance development to this compound**

Karolina Łuczkowska^1,§^, Katarzyna Ewa Sokolowska^2,§^, Olga Taryma-Lesniak^2^, Krzysztof Pastuszak^3^, Anna Supernat^3^, Jonas Bybjerg-Grauholm^5^, Lise Lotte Hansen^6^, Edyta Paczkowska^1^, Tomasz K. Wojdacz^2, 6, 7, *^, Bogusław Machaliński^1, *^

**Supplementary Material 1:** Lists of identified the DMRs (differentially methylated regions), hyper- and hypo methylated probes.

**Supplementary Material 2:** Outline of the experimental conditions and design details of the MS-HRM assays used in validation experiment.

**Table S1:**

Outline of the PCR and HRM conditions in validation experiments.

| Program | Cycles | Temperature (^o^C) | Acquisition Mode | Hold (sec) | Ramp Rate | Acquisitions (per ^o^C) |
| --- | --- | --- | --- | --- | --- | --- |
| Pre-Incubation | 1 | 95 | - | 600 | - | - |
| Amplification  Analysis mode: Quantification | 50 | 95 | None | 15 | 4,4 | - |
|  |  | *48 – 60 | None | 10 | 2,2 | - |
|  |  | 72 | Single | 15 | 4,4 | - |
| HRM  Analysis mode: Melting Curves | 1 | 95 | Continuous | 15 | 4,4 | - |
|  |  | 60 |  | 60 | 2,2 | - |
|  |  | 95 |  | - | 0,01 | 20 |

* Different for every MS-HRM assays, see description of the specific DMR

**Legend for the description of the assays:**

- Original sequence was translated to bisulfite modified sequence using: https://www.urogene.org/cgi-bin/methprimer/methprimer.cgi
- Red and **bold**- Primer binding sites (primers are included in length of the amplicons)
- 0% methylated DNA - 100% of probe contain unmethylated DNA
- 100% methylated DNA - 100% of probe contain methylated DNA
- 24h after lenalidomide treatment - DNA isolated from cell culture after 24 hours of incubation after lenalidomide treatment
- 10d after lenalidomide treatment - DNA isolated from cell culture after 10 days of incubation after lenalidomide treatment
- 24h after bortezomib treatment - DNA isolated from cell culture after 24 hours of incubation after bortezomib treatment
- 10d after bortezomib treatment – DNA isolated from cell culture after 10 days of incubation after bortezomib treatment
- 24h incubation of non-treated cells – DNA isolated from non-treated cell culture
- 10d incubation of non-treated cells – DNA isolated form non-treated cell culture

**DMR42**

**1. Methylation change observed on microarray: Loss**

**2. MS-HRM assay datils:**

chr6:33,288,281-33,288,400 (UCSC Genome Browser on Human Feb. 2009 (GRCh37/hg19) Assembly), Length: 89 bp

1 TGAGTGCTCTGACTTTATGTCTTCCCACGTAGGCGTTGACCCTGCACTATCAGATCCTGT

||||||:|:|||:|||||||:||:::|++||||++||||:::||:|:|||:||||::|||

1 TGAGTGTTTTGATTTTATGTTTTTTTAC**GTAGGCGTTGATTTTGTATTATTAGA**TTTTGT

61 GTTGGCCCGGCGCCTTCGGGAAAACCGGAGTTTGGCCATGAGTCGGCTGGATGAGGTCAT

|||||::++|++::||++||||||:++||||||||::||||||++|:||||||||||:||

61 GTTGGTTCGGCGTTTTCGGGAAAATCGGAGTTTGG**TTATGAGTCGGTTGGATGAGGTT**AT

Primer sequences:

F: **GTAGGCGTTGATTTTGTATTATTAGA**

R: **AACCTCATCCAACCGACTCATAA**

**3. MS-HRM results:**


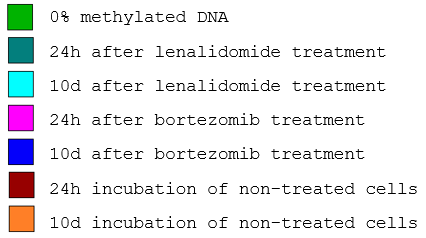

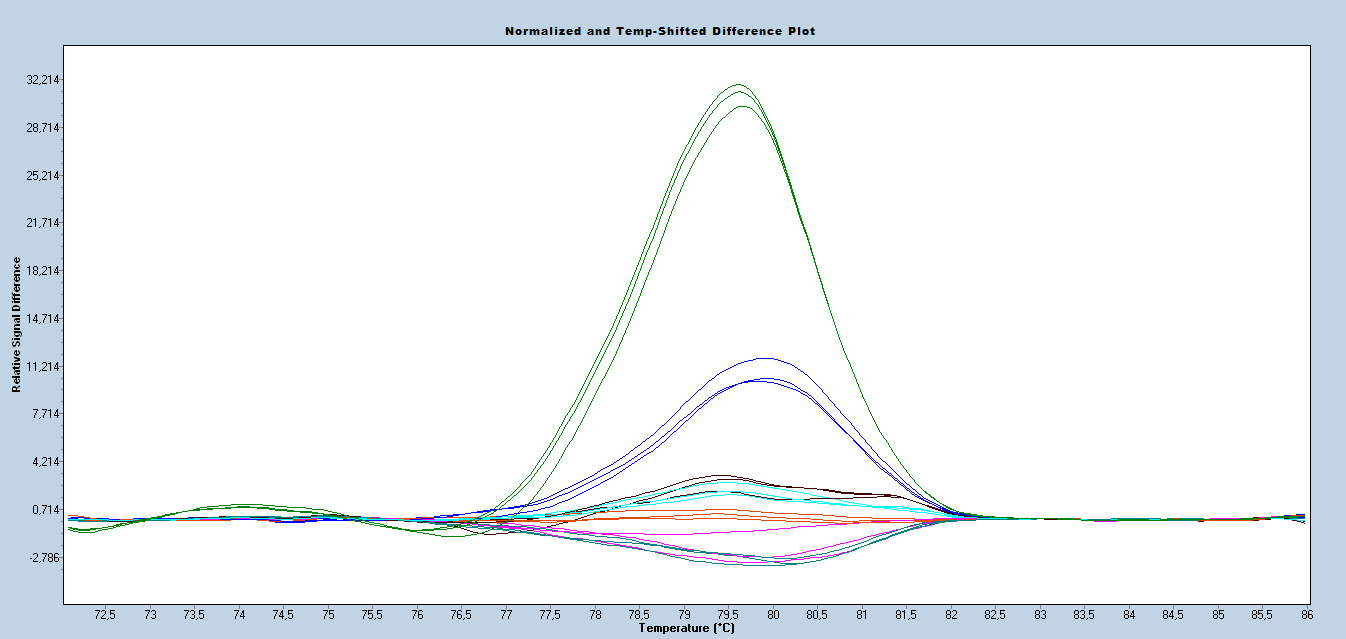


Specific PCR Conditions:

Mg 3mM, 60 deg.

**DMR37**

**1. Methylation change observed on microarray: Loss**

**2. MS-HRM assay datils:**

chr5:14,406,688-14,406,807 (UCSC Genome Browser on Human Feb. 2009 (GRCh37/hg19) Assembly), Length: 97 bp

1 AGAGGATCTGGACAGCCAAGGAGACGGCAGCAGCCAGCCTGATACGATTTCCATCGCCTC

|||||||:||||:||::|||||||++|:||:||::||::|||||++||||::||++::|:

1 AGAGGATTTGGATAGTTAA**GGAGACGGTAGTAGTTAGTTTGAT**ACGATTTTTATCGTTTT

61 ACGGACGTCTCAGAACACGCTGGACAGCGATAAGGTGAGTCACTGCCGGCACTTTGTGTG

|++||++|:|:||||:|++:||||:||++|||||||||||:|:||:++|:|:||||||||

61 ACGGACGTTTTAGAATACGTTGGATAGCGA**TAAGGTGAGTTATTGTCGGTATTTTG**TGTG

Primer sequences:

F: **GGAGACGGTAGTAGTTAGTTTGAT**

R: **CAAAATACCGACAATAACTCACCTTA**

**3. MS-HRM results:**


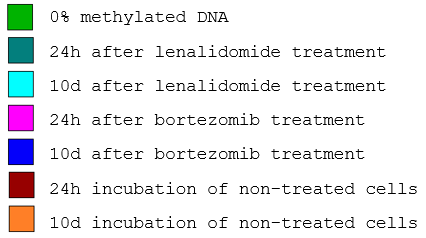

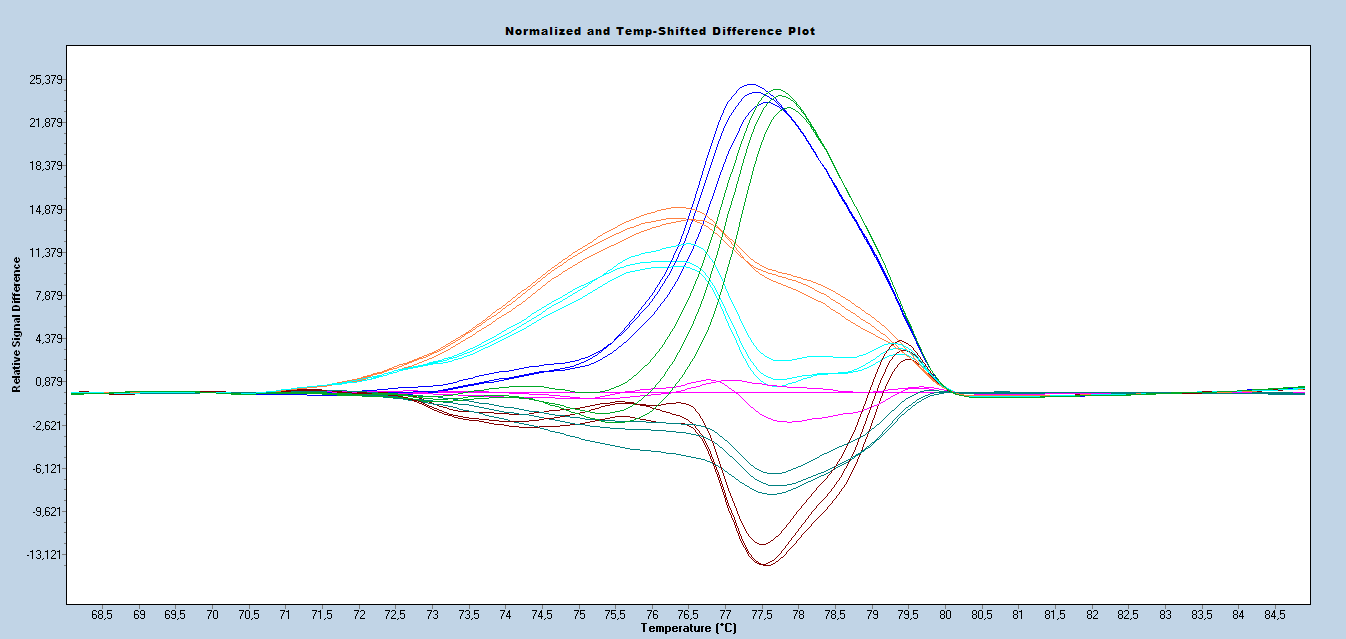


Conditions:

Mg 3mM, 58 deg.

**DMR6**

**1. Methylation change observed on microarray: Gain**

**2. MS-HRM assay datils:**

chr10:16,741,516-16,741,635 (UCSC Genome Browser on Human Feb. 2009 (GRCh37/hg19) Assembly), Length: 84 bp

1 ACACCACCAGAGCGCCGCTCAGCCTTCTAATAAAGGGGATGGGTGAGGGCTCCTAACTCA

|:|::|::||||++:++:|:||::||:||||||||||||||||||||||:|::|||:|:|

1 ATATTATTAGAGCGTCGTTTAGTTTTTTAA**TAAAGGGGATGGGTGAGGGTTTT**TAATTTA

61 ACGCACTGAAGCGTGTGGCGTCAGAGCGATTGCAGGCTGACTGACAGCGGGGTGAGATGC

|++:|:|||||++|||||++|:||||++||||:|||:|||:|||:||++||||||||||:

61 ACGTATTGAAGCGTGTGGCGTTAGAGCGATTG**TAGGTTGATTGATAGCGGGGTG**AGATGT

Primer sequences:

F: **TAAAGGGGATGGGTGAGGGTTTT**

R: **CACCCCGCTATCAATCAACCTA**

**3. MS-HRM results:**


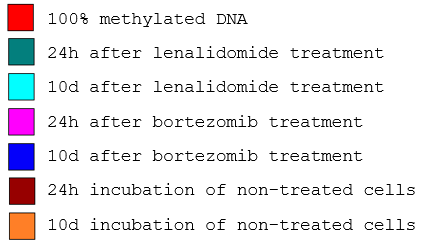

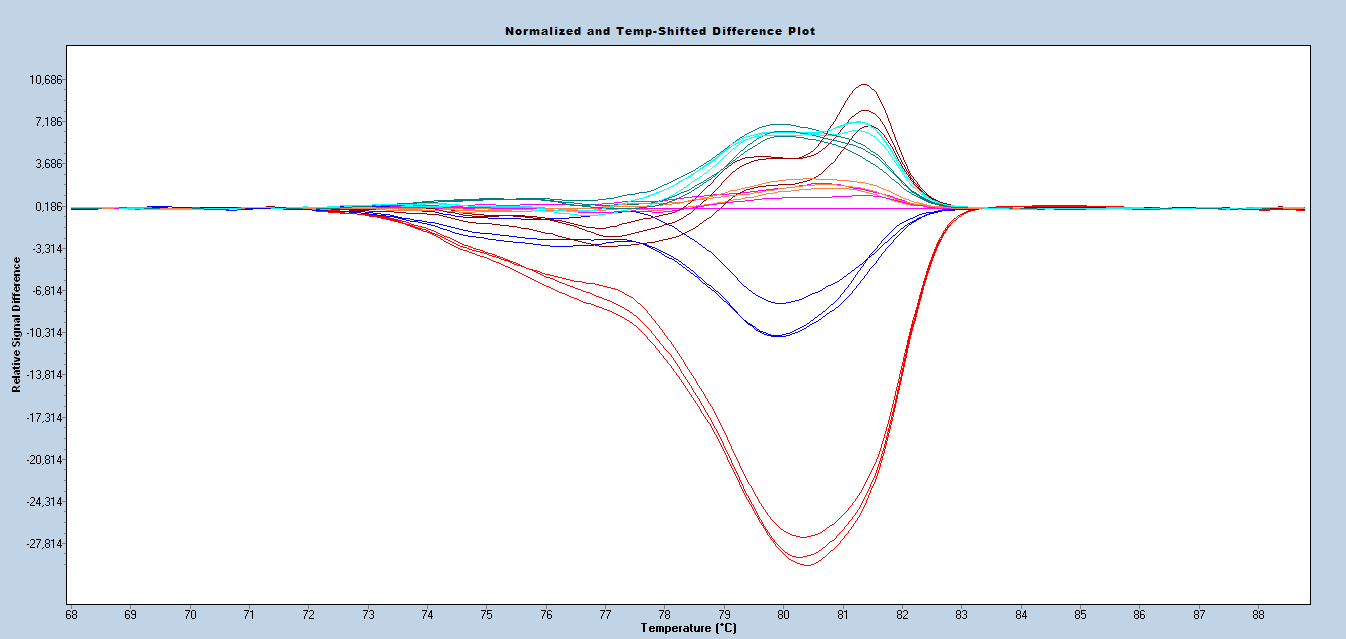


Conditions:

Mg 3mM, 58 deg.

**DMR29**

**1. Methylation change observed on microarray: Gain**

**2. MS-HRM assay datils:**

chr2:204,102,432-204,102,551 (UCSC Genome Browser on Human Feb. 2009 (GRCh37/hg19) Assembly), Length: 108 bp

1 AGCCAG**GCGAAATAGAAAGAGGGGTCC**TAGGGTATTTTATGCCCAGGGCAACAAACGCCA

||::|||++||||||||||||||||::||||||||||||||:::||||:||:|||++::|

1 AGTTAGGCGAAATAGAAAGAGGGGTTTTAGGGTATTTTATGTTTAGGGTAATAAACGTTA

61 AATGCTGACGTAGATAGCACTTATTCGGTTTTGCCAGGAGCTGTGCTAAGTGATTTTCAT

||||:|||++|||||||:|:|||||++||||||::|||||:||||:|||||||||||:||

61 AATGTTGACGTAGATAGTATTTATTCGGTTTT**GTTAGGAGTTGTGTTAAGTGAT**TTTTAT

Primer sequences:

F: **GGCGAAATAGAAAGAGGGGTTT**

R: **ATCACTTAACACAACTCCTAAC**

**3. MS-HRM results:**


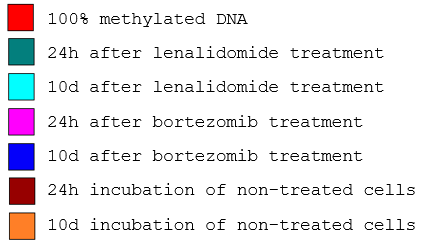

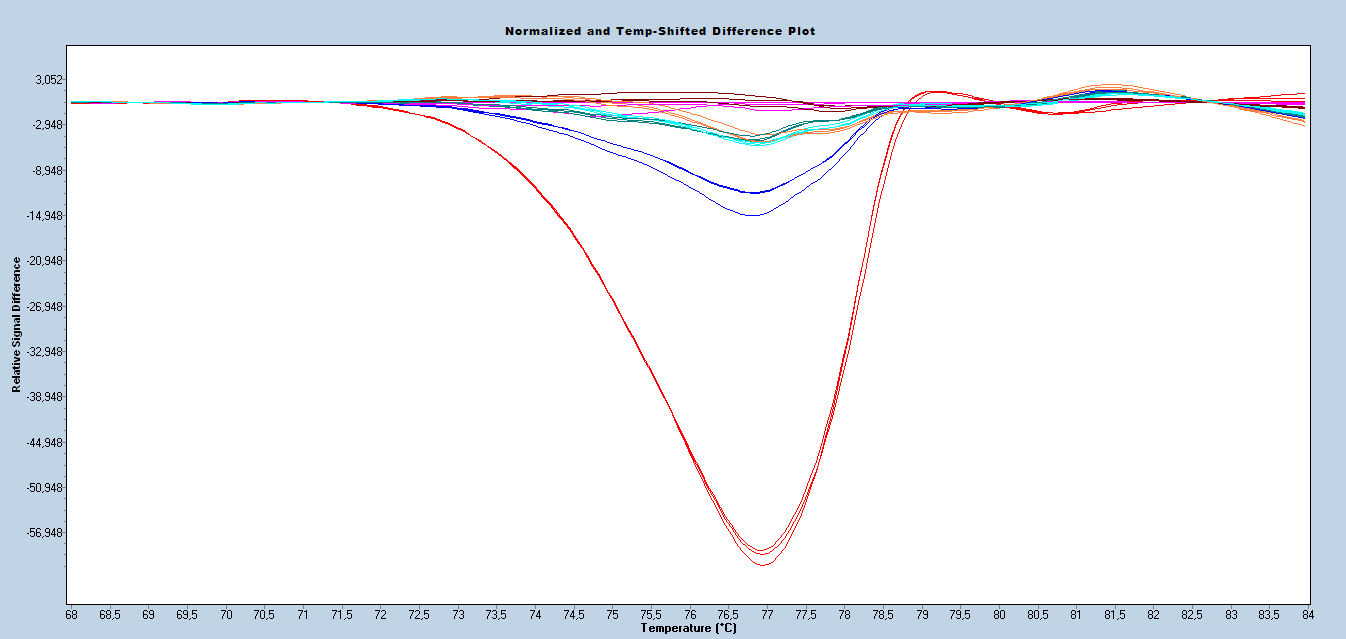


Conditions:

Mg 3mM, 48 deg.

**DMR43**

**1. Methylation change observed on microarray: Gain**

**2. MS-HRM assay datils:**

chr7:907,861-907,980 (UCSC Genome Browser on Human Feb. 2009 (GRCh37/hg19) Assembly), Length: 87 bp

1 CCCCCACCTCCAGGTGCAGGGCAGATGTGACCGGAGAGCACTGTTCGCAGCACAGCGTCC

:::::|::|::|||||:||||:||||||||:++|||||:|:||||++:||:|:||++|::

1 TTTTTATTT**TTAGGTGTAGGGTAGATGTGAT**CGGAGAGTATTGTTCGTAGTATAGCGTTT

61 CCAGGCACACACAGGGACTGACTGACAAGTTGCAGACGCTGCGCCCAGGCAGCCACGTTG

::|||:|:|:|:|||||:|||:|||:||||||:|||++:||++:::|||:||::|++|||

61 TTAGGTATATA**TAGGGATTGATTGATAAGTTGTAGA**CGTTGCGTTTAGGTAGTTACGTTG

Primer sequences:

F: **TTAGGTGTAGGGTAGATGTGAT**

R: **TCTACAACTTATCAATCAATCCCTA**

**3. MS-HRM results:**


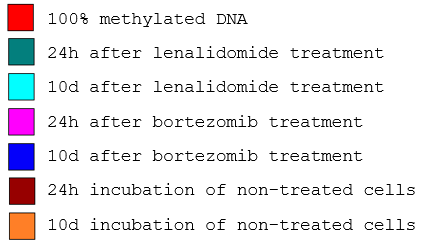

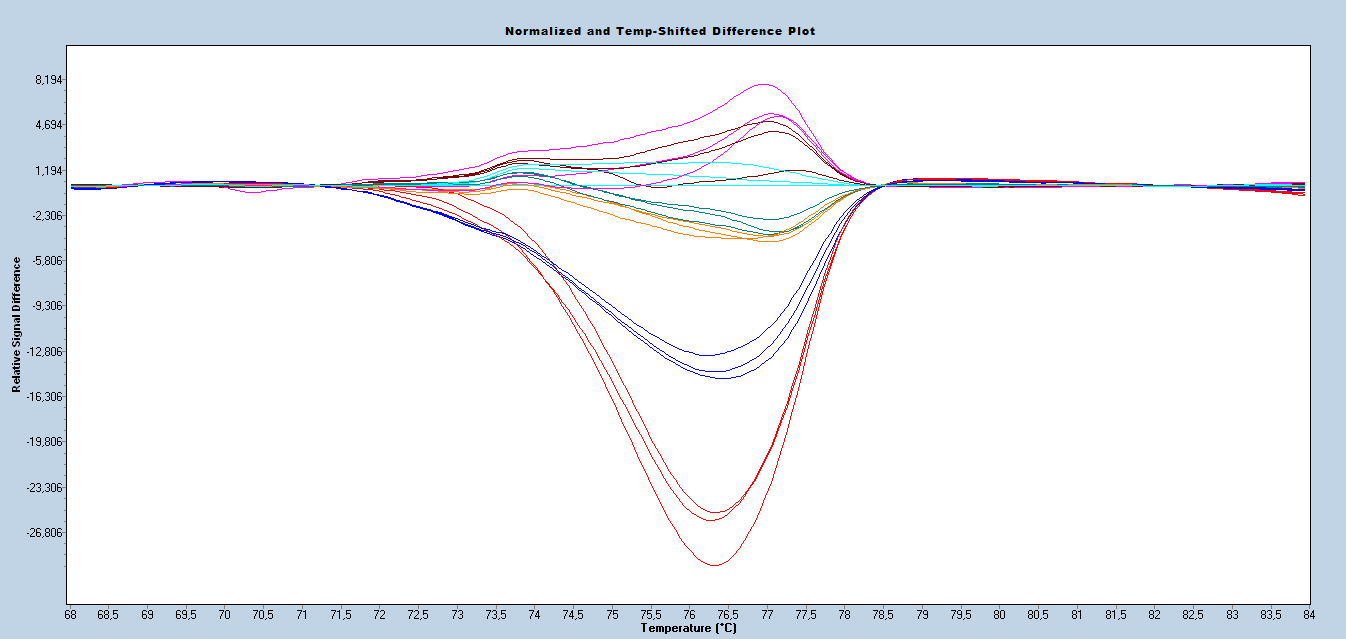


Conditions:

Mg 3mM, 54 deg.

**Figure S1.**

The schematic outline of the cell line experiment. The cells after each round of treatment (A) were let to recover for 12 days and re-treated (B). After each treatment the serving cells were re-seeded into sufficient number of the replicates (6 timepoints x 18 replicates) to allow reliable MTT-based (see methods for details) measurement of the proliferative potential at each of time point. Proliferative potential of the cells was tested after 0, 2, 5, 7, 9 and 12 days after each treatment. The procedure was repeated the second time (C).


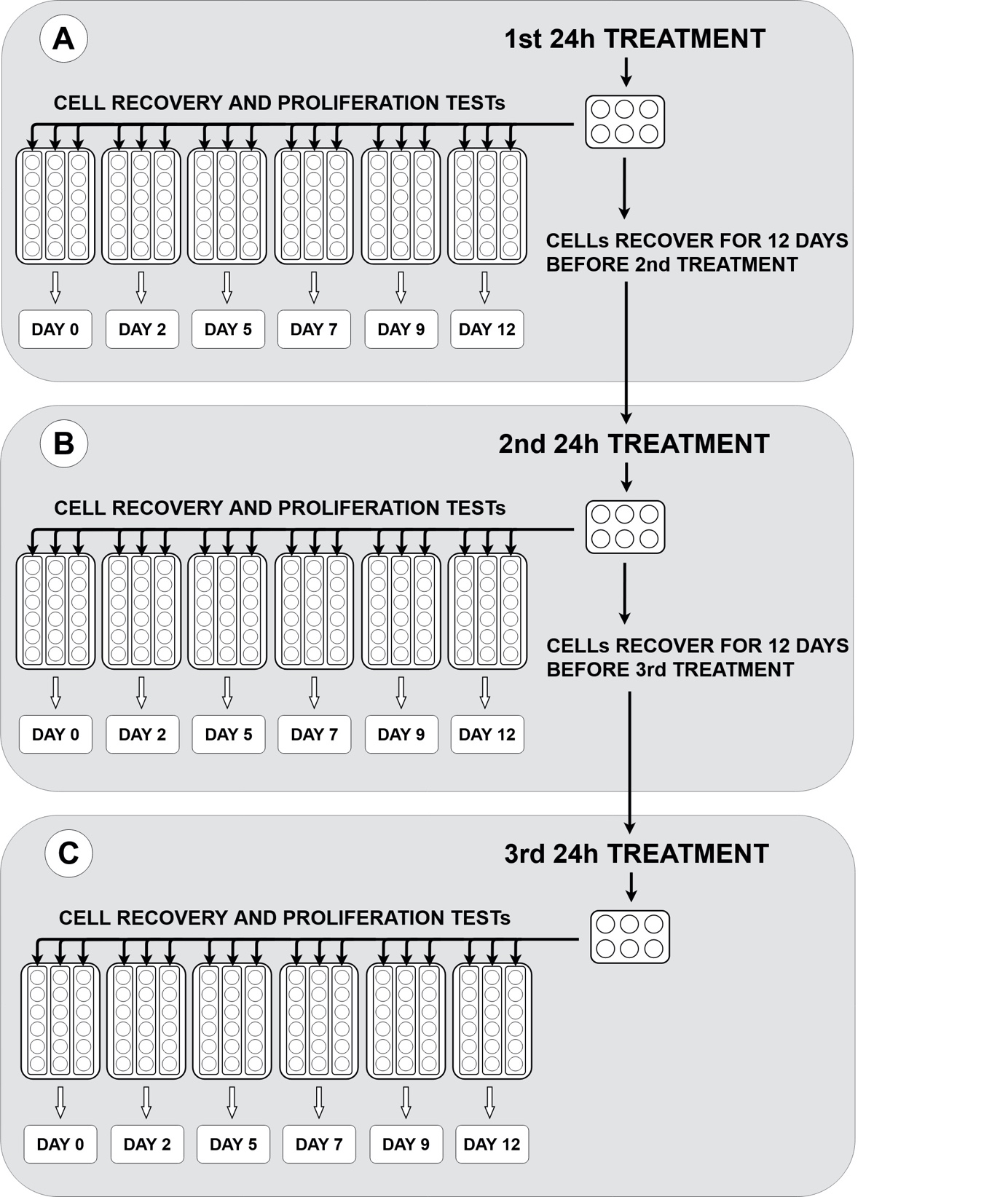


**Table S2.**

To identify *de novo* and known motifs we used HOMER (v4.11) (Heinz et al., 2010). Specifically, we used findMotifsGenome.pl with masking, hypergeometric enrichment calculations. We set an expected genome-wide distribution of EPIC probes as background. The size of the region used for motif finding was 200 bp. Below only results with equal and lower than 1e-12 p-value are presented (as HOMER recommended).

| **Rank** | **Motif** | **Score** | **p-value** | **% of Targets** | **% of Background** | **Best Match/Details** |
| --- | --- | --- | --- | --- | --- | --- |
| **1** | **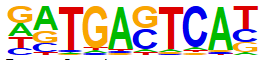** | **0.996** | **1e-447** | **29.00%** | **5.18%** | **JunB(bZIP)/DendriticCells-Junb-ChIP-Seq(GSE36099)/Homer** |
| **2** | **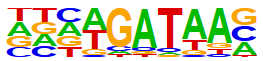** | **0.964** | **1e-83** | **29.97%** | **16.70%** | **GATA3(Zf)/iTreg-Gata3-ChIP-Seq(GSE20898)/Homer** |
| **3** | **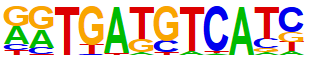** | **0.942** | **1e-49** | **5.26%** | **1.42%** | **CREB1/MA0018.4/Jaspar** |
| **4** | **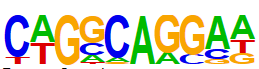** | **0.730** | **1e-32** | **32.68%** | **23.78%** | **NF1-halfsite(CTF)/LNCaP-NF1-ChIP-Seq(Unpublished)/Homer** |
| **5** | **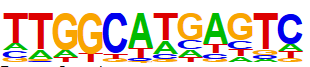** | **0.770** | **1e-28** | **2.60%** | **0.61%** | **NFIA/MA0670.1/Jaspar** |
| **6** | **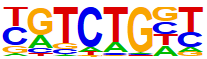** | **0.896** | **1e-25** | **22.01%** | **15.29%** | **Smad4(MAD)/ESC-SMAD4-ChIP-Seq(GSE29422)/Homer** |
| **7** | **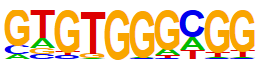** | **0.931** | **1e-25** | **6.03%** | **2.71%** | **Egr2(Zf)/Thymocytes-Egr2-ChIP-Seq(GSE34254)/Homer** |
| **8** | **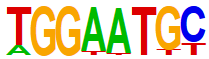** | **0.964** | **1e-21** | **8.80%** | **4.91%** | **TEAD3(TEA)/HepG2-TEAD3-ChIP-Seq(Encode)/Homer** |
| **9** | **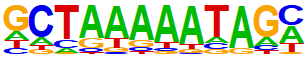** | **0.974** | **1e-20** | **2.29%** | **0.66%** | **Mef2d(MADS)/Retina-Mef2d-ChIP-Seq(GSE61391)/Homer** |
| **10** | **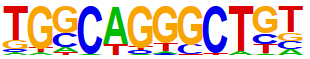** | **0.652** | **1e-19** | **5.46%** | **2.65%** | **ZNF449/MA1656.1/Jaspar** |
| **11** | **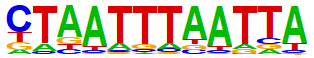** | **0.928** | **1e-17** | **12.65%** | **8.28%** | **PROP1/MA0715.1/Jaspar** |
| **12** | **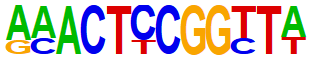** | **0.599** | **1e-16** | **0.25%** | **0.00%** | **SPDEF/MA0686.1/Jaspar** |
| **13** | **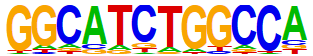** | **0.720** | **1e-13** | **2.63%** | **1.07%** | **HAND2/MA1638.1/Jaspar** |
| **14** | **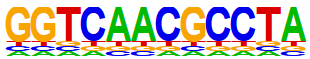** | **0.644** | **1e-13** | **0.17%** | **0.00%** | **RARg(NR)/ES-RARg-ChIP-Seq(GSE30538)/Homer** |
| **15** | **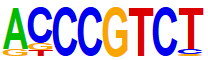** | **0.729** | **1e-13** | **2.35%** | **0.91%** | **Smad4(MAD)/ESC-SMAD4-ChIP-Seq(GSE29422)/Homer** |
| **16** | **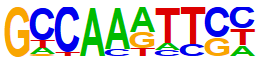** | **0.779** | **1e-13** | **2.83%** | **1.22%** | **NFIX/MA0671.1/Jaspar** |
| **17** | **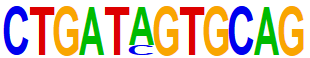** | **0.567** | **1e-12** | **0.20%** | **0.00%** | **ZSCAN22(Zf)/HEK293-ZSCAN22.GFP-ChIP-Seq(GSE58341)/Homer** |
